# Severe abdominopelvic actinomycosis with colon perforation and hepatic involvement mimicking advanced sigmoid colon cancer with hepatic metastasis: a case study

**DOI:** 10.1186/s12893-018-0386-3

**Published:** 2018-08-02

**Authors:** Song Soo Yang, Yeong Cheol Im

**Affiliations:** Department of Surgery, Ulsan University Hospital, University of Ulsan College of Medicine, 877, Bangeojinsunhwando-ro, Dong-gu, Ulsan, 44033 Republic of Korea

**Keywords:** Abdominopelvic actinomycosis, Colon perforation, Liver involvement

## Abstract

**Background:**

Actinomycosis is a rare chronic invasive disease caused by *Actinomyces* spp. Although abdominopelvic actinomycosis, which involves the colon and the pelvic organs extensively, has been frequently reported, abdominopelvic actinomycosis presenting with colon perforation and hepatic involvement concurrently has yet to be reported.

**Case presentation:**

A 55-year-old woman presented at the emergency room with squeezing epigastric pain. Palpation of the abdomen revealed a hard mass with no acute peritoneal signs. Vital signs were normal range except for tachycardia. Initial laboratory testing revealed leukocytosis, anemia, elevated C-reactive protein (CRP), hypoalbuminemia; and normal AST/ALT and BUN/creatinine. CT scan of the abdomen-pelvis revealed a microperforations of the sigmoid colon, abscess in the left lower quadrant and hepatic lesion. Furthermore, there was a large infiltrating conglomerated mass invading the urinary bladder, left adnexa, sigmoid, left inguinal canal and left pelvic wall area. Ultrasound revealed an intra-uterine device (IUD). All these findings initially raised a suspicion of malignancy such as advanced cancer of the colon with liver metastasis. Despite the rarity of the disease, actinomycosis were not excluded because of the IUD found on ultrasound. Parenteral antibiotics and percutaneous drainage of abdomen abscess as well as fasting with total parental nutrition were prescribed for sigmoid perforation and abscess. After 10 days of conservative treatment, no remarkable change was detected in conglomerated mass invading pelvis. Furthermore, the finding of newly developed mechanical small bowel obstruction warranted surgery. Exploratory laparotomy was performed for the removal of perforated colon, obstructive small bowel and organs involved and postoperative histology confirmed a diagnosis of colonic actinomycosis. The patient made an uneventful recovery and was started on a 6-month course of penicillin.

**Conclusions:**

Abdominopelvic actinomycosis presenting with colon perforation and hepatic involvement is extremely rare; however, it is clinically similar to advanced colon cancer with liver metastasis, therefore, complicating the preoperative diagnosis. A diagnosis of abdominopelvic actinomycosis should be considered in patients with a history of IUD and chronic abdominal pain, along with an abdominal mass or cutaneous abscess. If surgery is indicated, preoperative empirical antibiotic therapy for actinomycosis and frozen biopsy during surgery may be considered.

## Background

Actinomycosis is a rare chronic invasive disease and *Actinomyces israelii* is the most prevalent species, anaerobic gram-positive bacteria that normally colonize oral, digestive and urogenital tracts in humans [1]. Breach of tissue integrity in mucosal lesions facilitates invasion of local structures and organs, leading to pathogenic co-infection with other organisms. All the tissues and organs may be infected, but four main clinical types of infection can be distinguished, depending on the primary site of infection: cervicofacial, thoracic, abdominopelvic, and disseminated disease [2]. Abdominopelvic actinomycosis is a rare disease encompassing abdominal infection, intrauterine devices (IUD)-related pelvic abscesses, infections of appendix, rectum and liver [3]. When it is associated with gastrointestinal organs, it is similar to chronic inflammatory bowel disease or malignancy, especially colon cancer [4]. Although abdominopelvic actinomycosis, which involves the colon and the surrounding pelvic organs extensively, has been frequently reported, abdominopelvic actinomycosis presenting with colon perforation and hepatic involvement concurrently has yet to be reported.

Here, we report a severe case of abdominopelvic actinomycosis with sigmoid colon perforation and hepatic lesion mimicking advanced colon cancer with liver metastasis.

## Case presentation

A 55-year-old woman with no specific medico-surgical history presented at the emergency room with a 1-day history of squeezing epigastric abdominal pain. Patient also complained of a thick turbid yellowish discharge in the left inguinal area that was intermittently drained for some years.

Vital signs were normal range except for tachycardia (pulse rate, 110/min). Palpation of the abdomen revealed a wood-like hard mass in the left lower quadrant with minimal tenderness and no acute peritoneal signs warranting emergent surgery. A visible scar was noted in the left inguinal area without any discharge.

**Fig. 1:**
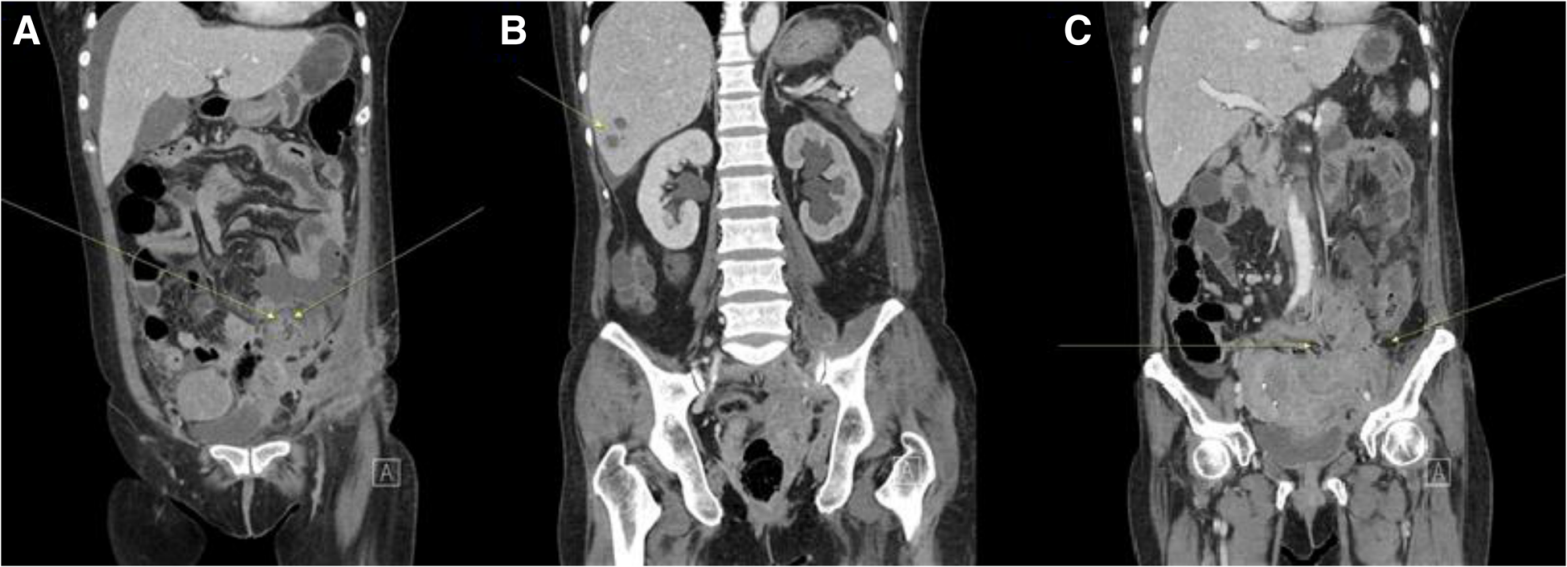
CT finding at the emergency room. **a** CT scan of the abdomen-pelvis revealed a microperforation (*arrow*) of the sigmoid colon and abscess in the left lower quadrant. **b** CT scan showed a hepatic lesion (*arrow*) and bilateral hydronephrosis. **c** There was a large infiltrating heterogenous hyperattenuating conglomerated mass invading the urinary bladder, left adnexa, sigmoid, left inguinal canal and left pelvic wall area (*arrow*)

**Fig. 2:**
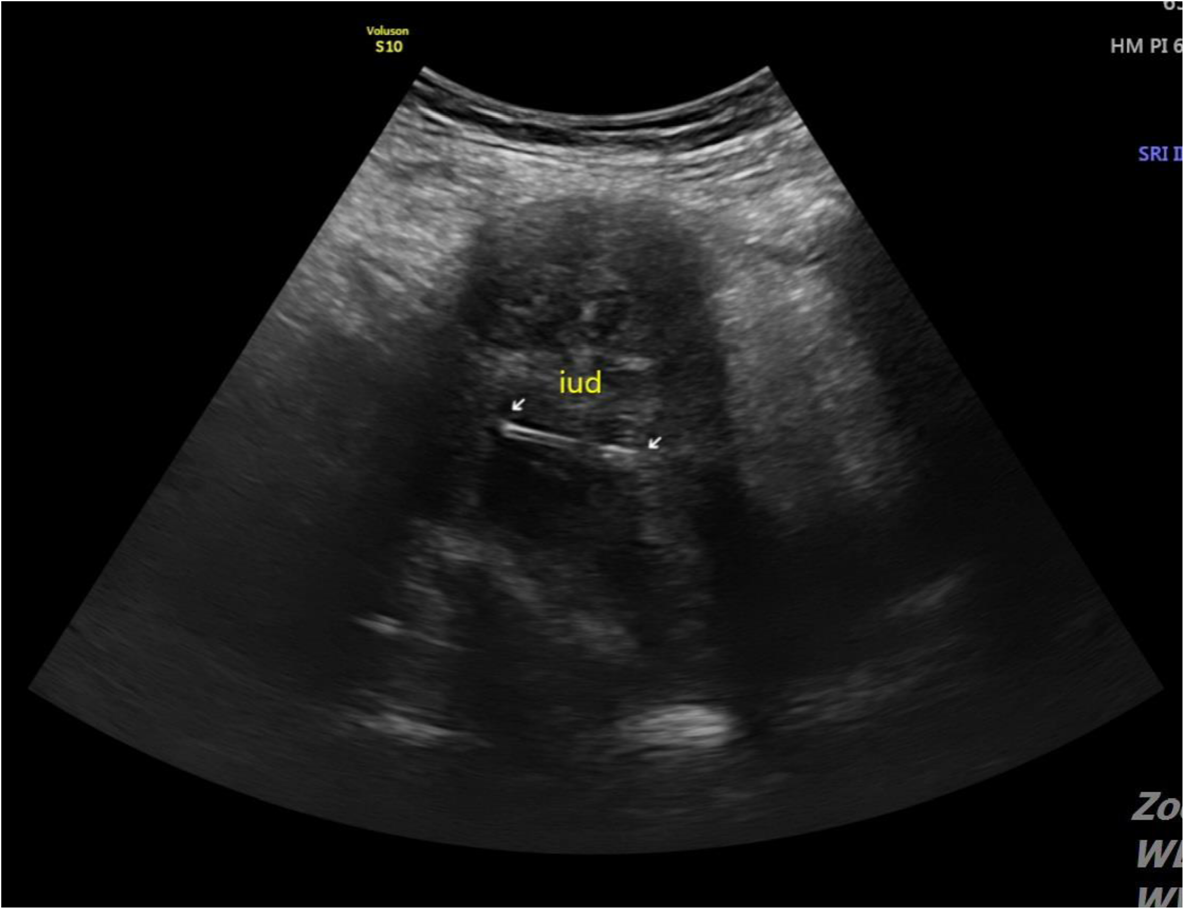
Ultrasound revealed an intra-uterine device (IUD)

Initial laboratory testing revealed marked leukocytosis (white blood cells, 24,730 cells/mm^3^), anemia (hemoglobin concentration of 6.9 g/dL), elevated C-reactive protein (CRP) 32.05 mg/dL (reference range, 0–0.5 mg/dL), hypoalbuminemia (albumin, 2.5 g/dL); and normal AST/ALT and BUN/creatinine. CT scan of the abdomen-pelvis revealed a microperforation of the sigmoid colon, abscess in the left lower quadrant, a hepatic lesion and bilateral hydronephrosis. Furthermore, there was a large infiltrating heterogenous hyperattenuating conglomerated mass invading the urinary bladder, left adnexa, sigmoid, left inguinal canal and left pelvic wall area (Fig. 1). Ultrasound revealed an intra-uterine device (IUD) (Fig. 2). All these findings initially raised a suspicion of malignancy such as advanced cancer of the colon or ovary with liver metastasis. Despite the rarity of the disease, infectious diseases such as actinomycosis were not excluded because of the IUD found on ultrasound. Colonoscopy or percutaneous needle biopsy was not performed for accurate diagnosis due to suspected colon perforation and the small bowel enclosed mass.

Since the patient showed minimal peritoneal irritation and stable vital signs, and extensive organ resection was expected due to invasion of bladder and ureters, treatment was initially conservative rather than primary debulking surgery. The antibiotic regimen was always determined based on the infectious disease diagnosis after hospitalization. Parenteral antibiotics (ceftriaxone+metronidazole+azithromycin) and fasting with total parental nutrition were prescribed for sigmoid perforation. Because there was a huge left abdominal abscess (11X8X3cm) that could spread to other spaces and cause generalized peritonitis, the imaging-guided percutaneous abscess drainage was performed.

After 10 days of conservative treatment, a repeat CT scan of the abdomen-pelvis showed improvement in abdominal abscess and liver lesion. However, no remarkable change was detected in conglomerated mass invading pelvis. Furthermore, the finding of newly developed mechanical small bowel obstruction warranted surgery.

Exploratory laparotomy was performed for the removal of perforated colon, obstructive small bowel and organs involved. Abscess of the sigmoid colon involved the uterus, adnexa, loop of small bowel and distal colon with severe adhesion between the mass and pelvic organs including the uterus, small and large bowels, and bladder. The abscess compressed the left ureter and caused ureteral dilatation. En-bloc excision of the mass was performed using Hartmann’s procedure, bilateral salpingo-oophorectomy, small bowel resection and appendectomy. The gynecologist decided not to resect uterus because of severe fibrotic adhesion in the pelvis and transvaginal IUD removal failed repeatedly due to severe adhesion.

**Fig. 3:**
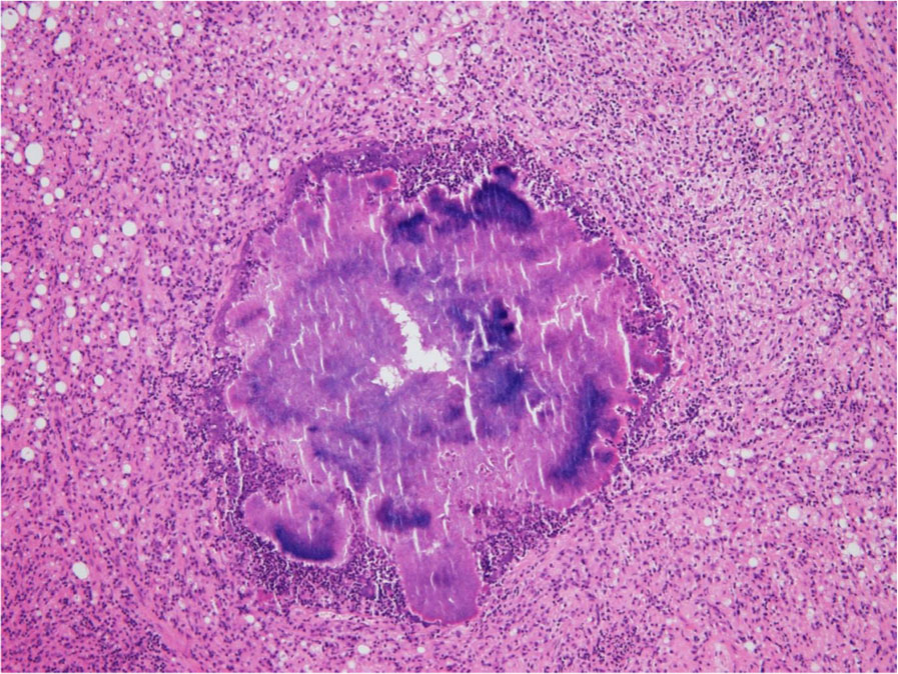
This histologic section showed the granular colonies of bacteria, commonly termed sulfur granule, with aggregates of filamentous bacteria and neutrophils. H&E × 100

Since the frozen section excluded malignancy, a double J catheter was inserted into both the ureters without resection. Although *Actinomyces* spp. failed to grow in preoperative cultures, postoperative permanent histology confirmed a definitive diagnosis of colonic actinomycosis, which showed the granular colonies of bacteria, commonly termed sulfur granule, with aggregates of filamentous bacteria and neutrophils (Fig. 3) and abscess with invasion into the uterus and ovaries.

After surgery, the parenteral antibiotic regimen was changed to tigecycline, amikacin, metronidazole and Penicillin G. Three days after surgery, bowel movement was restored and vital signs were stabilized, which decreased the abdominal pain. The patient made an uneventful recovery and was started on a 6-month course of penicillin. At 1-year follow-up, the patient was well and free from disease.

## Discussion and conclusions

Abdominopelvic actinomycosis is one of the main clinical types according to the site of infection. It is a rare disease, but it leads to tissue granulation, dense fibrosis and abscess, resulting in a hard pelvic mass compressing the urinary bladder, ureter and rectum [5]. Previous studies of actinomycosis showed bowel strictures or hydronephrosis [6, 7]. However, whatever the origin of infection, colon perforation is a rare event. There is a case that a single perforation of the transverse colon at the hepatic flexure caused by obstructing sigmoid colon mass was managed by emergency surgery and subsequent histologic examination revealed actinomycosis [8]. In our case, the precise factors underlying spontaneous colon perforation are unclear. However, it is assumed that the high pressure of the proximal colon due to the colorectal stricture as well as persistent inflammation of the sigmoid colon wall due to actinomycosis triggered colon perforation.

Hepatic actinomycosis (HA) is also a very rare form of abdominal actinomycosis and often, it is a secondary infection following abdominal infection. HA constitutes 15% of abdominal actinomycosis, and 5% of all actinomycosis [9–11]. The clinical features, diagnosis and treatment of colonic and hepatic actinomycosis in previous cases were shown in Table 1.

**Table 1:** Overview of previous reported case of colonic and hepatic actinomycosis

Reference	Year	Total cases	Involved sites	Mean age	Gender	Symptoms	IUD	Leukocytosis	Anemia	Presumptive diagnosis	Confirmatory test	Treatment
Colonic actinomycosis												
4	1995	1	Left colon	41	Female	Abdominal pain, nausea, constipation	+	–	+	Tumor of colon or retroperitoneum	Histologic diagnosis of surgical specimen	Complete excision of mass with colectomy + actinomycosis medication
30	2000	1	Transverse colon	37	Female	Abdominal pain with a sensation of fullness	–	–	+	Colon cancer	Histologic diagnosis of surgical specimen	Colectomy + actinomycosis medication
2	2000	4	Sigmoid, rectum	48.8 (38–55)	Male: 2 case Female: 2 case	Abdominal pain, constipation, weight loss, indigestion	+(1 female)	All case: +	n.av	colorectal cancer	Histologic diagnosis of surgical specimen: 3 case Biopsy: 1 case	3 case: colectomy + actinomycosis medication 1 case: actinomycosis medication
19	2000	1	Rectosigmoid, right colon, uterus, adnexa,	49	Female	Abdominal pain, constipation, vomiting	+	n.av	n.av	Colon cancer	Histologic diagnosis of surgical specimen	Colectomy + ovary excision + actinomycosis medication
28	2000	1	Rectosigmoid, right ureter	63	Male	Abdominal pain, constipation, malaise, weight loss	n.ap	+	–	colon cancer	Biopsy	Diverting sigmoidostomy + ureteral stent + actinomycosis medication
29	2002	1	Sigmoid, uterus, adnexa, right ureter	63	Female	Abdominal pain, fever	+	+	–	Pelvic actinomycosis or malignancy	Histologic diagnosis of surgical specimen	Total hysterectomy, bilateral salphigo-oophorectomy, adhesiolysis around urter + actinomycosis medication
8	2004	1	Sigmoid	39	Male	Abdominal pain	n.ap	+	n.av	Colon perforation due to obstructing colon cancer	Histologic diagnosis of surgical specimen	Colectomy, ileostomy + actinomycosis medication
31	2006	1	Sigmoid, both adnexa	38	Female	Abdominal pain, constipation, fever	+	+	n.av	Crohn;s disease or sigmoid tumor	Histologic diagnosis of surgical specimen	Colectomy + bilateral salphingo-oophorectomy + actinomycosis medication
18	2008	1	Rectosigmoid, uterus, adnexa, left ureter	42	Female	Pelvic discomfort, constipation	+	–	+	Advanced Ovarian cancer	Histologic diagnosis of surgical specimen	Neoadjuvant chemotherapy +total hysterectomy, bilateral salphingo-oophorectomy, rectosigmoid resection + ureteral stent + actinomycosis medication
Hepatic actinomycosis												
20	1997	11	Right lobe: 1 case Left lobe: 2 case Central area: 1 case No data: 7 case	55 (20–86)	Male: 7 case Female: 4 case	Fever: 9/11 (81.9%) Abdominal pain: 6/11 (54.5%) Palpable mass: 4/11 (36.4%) Back pain: 2/11 (18.2%) Nausea, vomiting: 2/11 (18.2%) Anorexia: 1/11 (9%) Diarrhea: 1/11 (9%) No clinical sign: 1/11 (9%)	n.av	+: 7/9 (77.8%)	+: 2/9 (22.2%)	Liver tumor: 6/11 (54.5%) Liver abscess: 5/11 (45.5%)	Histologic diagnosis of surgical specimen: 6/11 (54.5%) Biopsy: 1/11 (9%) Sulfur granule in pus: 2/11 (18.2%) Actinomyces culture: 2/11 (18.2%)	Liver resection + actinomycosis medication: 5/11 (45.5%) Surgical or percutaneous drainage + actinomycosis medication: 5/11 (45.%) Liver resection only: 1/11 (9%)
11	2010	1	Liver (both lobe)	70	Male	Fever, abdominal pain, anorexia, weight loss	n.ap	+	+	Hepatic metastasis	Biopsy	Actinomycosis medication
10	2011	1	Right lobe, single lesion	65	Male	Incidental finding of regular surveillance after pancreatic adenocarcinoma	n.ap	–	n.av	Metastatic liver tumor	Histologic diagnosis of surgical specimen	Liver resection + actinomycosis medication
9	2011	1	Liver (right lobe), ovary	41	Female	Abdominal pain	+	+	+	Ovarian cancer with hepatic metastasis	Histologic diagnosis of surgical specimen	Rt. Salpingo-oophorectomy + IUD removal + acitomycosis medication
3	2012	1	Liver(multiple nodules on surface), spleen	37	Male	Fever, abdominal pain	n.ap	+	–	Spleen abscess	Histologic diagnosis of surgical specimen	Splenectomy + liver biopsy + actinomycosis medication
13	2014	1	Left lobe	55	Male	Abdominal pain, weight loss	n.ap	–	–	Liver tumor	Histologic diagnosis of surgical specimen	Liver resection + actinomycosis medication
		32 (data of analysis in literature)	Right lobe: 19/29 (65.5%) Both lobe: 8/29 (27.6%) Left lobe: 2/29 (6.9%)	45.5 (5–86)	Male: 19 (59%) Female: 13 (41%)	Fever: 25/27 (92.6%) Weight loss: 15/25 (60%)	2 (6.3%)	+: 27/29 (93.1%)	+: 17/24 (70.8%)	Liver tumor: 20/28 (71.4%) Hepatophyta: 8/28 (28.6%) Liver hydatidosis: 2/28 (7.1%) Inflammatory pseudotumor: 1/28 (3.65%) Tuberculosis: 1/2 (3.65%)	Gram staining: 22/27 (81.5%) Histologic diagnosis of sulfur granule: 22/31 (71%) Actinomyces culture: 10/20 (50%)	Only actinomycosis medication: 14/32 (43.8%) Surgical or percutaneous drainage + actinomycosis medication: 12/32 (37.5%) Liver resection + actinomycosis medication: 6/32 (18.7%)

*n.av.* not available, *n.ap* not applicable

In the present unusual case, the patient presented with signs and symptoms mimicking advanced colon cancer with liver metastasis. Few studies have demonstrated other clinical features such as mimicking colon cancer, large mass with perforation, hydronephrosis and involvement of adjacent tissues. However, there are no reports showing all the features including colon perforation, rectal stricture, hydronephrosis, ascites and hepatic involvement.

Abdominopelvic actinomycosis does not produce the characteristic disease signs or symptoms and usually manifests as a slowly growing mass, which may be associated with altered bowel habits, nausea, vomiting and cramping pain [12]. Patients with hepatic involvement present mostly with chronic or subacute and non-specific symptoms including anorexia, weight loss, fever and night sweats [11, 13]. The nonspecific findings complicate the differential diagnosis of abdominopelvic actinomycosis from other chronic diseases such as chronic granulomatous infection, inflammatory bowel disease, and pelvic inflammatory diseases. Furthermore, it often presents as a mass either clinically or radiologically, which is not easily distinguishable from malignancies [12, 14, 15].

Several reports show abdominopelvic actinomycosis mimicking malignancies [16, 17]. Due to the misdiagnosis, several previous cases were treated with neoadjuvant chemotherapy [17–19]. Hepatic actinomycosis is also difficult to distinguish from primary hepatocellular carcinoma and metastatic liver cancer [20].

In addition, because the preoperative diagnosis is rarely considered and is established only in less than 10% of cases, the diagnosis is based on clinical manifestations and imaging findings [12, 21]. Since the cultures of *Actinomyces* species show very low yield, histopathological examination is the most utilized diagnostic method worldwide, which is generally conducted after surgical intervention due to an initial diagnostic error [4, 12, 22]. Sulfur granules were observed in the purulent material in 50% of cases. Although these might not be pathognomonic of actinomycosis, the presence of sulfur granules is highly suggestive of the diagnosis [23, 24].

In our case, colon perforation with liver lesion resembled advanced colon cancer with liver metastasis. Radiologically, actinomycosis was considered, but colon cancer with liver metastasis was not excluded.

Treatment of abdominopelvic actinomycosis depends on the extent of the disease and the patient’s condition. Long-term treatment with penicillin is the standard medical therapy for uncomplicated cases [25]. Indeed, *Actinomyces* spp. are usually extremely susceptible to beta-lactams, and especially Penicillin G or amoxicillin. Clindamycin, tetracycline, and erythromycin are alternatives in cases of allergy to penicillin [12]. Piperacillin-tazobactam or a carbapenem (imipenem or meropenem) may be an appropriate alternative [26]. The need for surgery must be assessed on an individual basis and surgery may be a valid option for patients who do not respond to medical treatment [26].

Treatment of HA mainly involves surgical or puncture drainage, hepatic resection, and postoperative treatment with anti-infectives [9, 20].

This particular case is interesting in several aspects.

First, although preoperative antibiotic therapy was administered only for 2 weeks, a significant reduction in inflammation due to actinomycosis was detected during surgery. The bladder and ureter, which were expected to be sacrificed, were preserved. Ureteral dilatation and hydronephrosis were resolved following insertion of a temporary double-J stent, and antibiotic therapy as reported previously [27–29].

Second, the exclusion of malignant tumors in frozen biopsy during surgery also facilitated the demarcation of the extent of surgery. In most of the previous cases, abdominopelvic mass was considered as a malignant tumor before surgery, and the diagnosis of actinomcycosis was made after surgery [2, 4, 8, 18, 19, 28–31]. However, in our case, by excluding the malignant tumor through the frozen biopsy during surgery, we could avoid unnecessary extensive surgery.

Therefore, we recommend the use of preoperative empirical antibiotics and exclusion of malignant tumors during surgery via frozen biopsy. Such a strategy reduces the extent of surgery and postoperative complications in patients, with actinomycosis indistinguishable from malignant tumor before surgery.

In conclusion, abdominopelvic actinomycosis presenting with colon perforation and hepatic involvement is extremely rare; however, it is clinically similar to advanced colon cancer with liver metastasis, therefore, complicating the preoperative diagnosis. A diagnosis of abdominopelvic actinomycosis should be considered in patients with a history of IUD and chronic abdominal pain, along with an abdominal mass or cutaneous abscess. If surgery is indicated, preoperative empirical antibiotic therapy for actinomycosis and frozen biopsy during surgery may be considered.

## Author’s ex-post considerations

- A diagnosis of abdominopelvic actinomycosis should be considered in patients with a history of IUD, even though concurrent hepatic mass was detected.
- If the patient’s condition allows, the use of preoperative empirical antibiotics should be considered for at least 2 weeks to decrease the extent of surgery and postoperative complications.
- If surgery is indicated, exclusion of malignant tumors via intraoperative frozen biopsy facilitated the determination of the extent of surgery.

## Abbreviations

ALT: Alanine transaminase
AST: Aspartate transaminase
BUN: Blood urea nitrogen
CRP: C-reactive protein
CT: Computer tomography
HA: Hepatic actinomycosis
IUD: Intra-uterine device
